# Quality of life and utility decrement associated with *Clostridium difficile* infection in a French hospital setting

**DOI:** 10.1186/s12955-019-1081-5

**Published:** 2019-01-11

**Authors:** Frédéric Barbut, Tatiana Galperine, Philippe Vanhems, Alban Le Monnier, Bernard Durand-Gasselin, Frédérique Canis, Viviane Jeanbat, Anne Duburcq, Sarah Alami, Caroline Bensoussan, Francis Fagnani

**Affiliations:** 1 National Reference Laboratory for Clostridium difficile, Hôpital Saint-Antoine, 34 rue Crozatier, 75012 Paris, France; 2 Université Paris Descartes, UMR-S1139, Sorbonne Paris Cité, Paris, France; 3 CHRU Lille, Maladies Infectieuses, French Group of Faecal Microbiota Transplantation (GFTF), Lille, France; 4 Groupement Hospitalier Edouard Herriot, Unité d’Hygiène, Epidémiologie et Prévention, Hospices Civils de Lyon, and Université Lyon 1, Lyon, France; 5 Laboratoire de Microbiologie Clinique, GH Paris Saint-Joseph, Paris, France; 6 Hôpital Léopold Bellan, Service de gériatrie and Fondation Hospitalière Ste Marie Service de Soins de Suite et de Réadaptation gérontologique, Paris, France; 7 Centre hospitalier de Valenciennes, UF de Microbiologie, Pôle de Biologie Médicale, Valenciennes, France; 8 CEMKA, Bourg la Reine, France; 9 MSD France, Courbevoie, France

**Keywords:** Clostridium difficile, Health-related quality of life, EQ-5D, France

## Abstract

**Background:**

*Clostridium difficile* infection (CDI) is associated with a substantial Quality of life impact on patients that has not been so far measured with a generic validated instrument.

**Methods:**

A prospective study was performed in 7 French acute-care settings in patients presenting with a bacteriologically-confirmed CDI. The EQ-5D-3 L was filled in by patients at 7 ± 2 days after CDI diagnosis to describe their state of health at that date as well as their state of health immediately before the CDI episode (baseline). Individual utility decrement was obtained by subtracting the corresponding utilities. The Quality Adjusted Life Year (QALY) loss was calculated by multiplying the days spent from baseline to the date of the interview, by the decrement of utility. A multivariate analysis of variance of the utility decrement according to CDI and patients characteristics was performed.

**Results:**

Eighty patients were enrolled (mean age: 69.4 years, 55% females). The utility scores dropped from a mean 0.542 (SD: 0.391) at baseline to 0.050 (SD: 0.404) during the CDI episode with a mean adjusted utility decrement of 0.492 (SD: 0.398) point. This decrement increased significantly with CDI severity (Zar score ≥ 3) (*p* = 0.001), in patients with a positive baseline utility (*p* = 0.032), in women as compared to men (*p* = 0.041) and in patients aged more than 65 years (*p* = 0.041). No association with the Charlson index was found. The associated QALY loss not integrating the excess mortality was 0.028 (SD: 0.053).

**Conclusions:**

The impact on quality of life of CDI episodes is major and translates in a substantial QALY loss despite their short duration.

**Electronic supplementary material:**

The online version of this article (10.1186/s12955-019-1081-5) contains supplementary material, which is available to authorized users.

## Background

Clostridium difficile is an anaerobic Gram-positive bacterium responsible for 15–25% of cases of post-antibiotic diarrhea and virtually all cases of pseudomembranous colitis [1, 2]. It is also the main causative agent of nosocomial diarrhea among hospital inpatients [3]. Clinically, *C. difficile* infection (CDI) ranges from uncomplicated diarrhea to fulminant, and sometimes fatal, pseudomembranous colitis. The estimated all-cause 30-day mortality rate among patients who acquire CDI in healthcare facilities is between 3 and 30% overall [4, 5].

Different therapeutic options are already available and new treatments are currently in development raising the question of defining the best strategies for managing this infection at first episode as well as further recurrences. For this purpose, various economic studies have been performed to document alternative strategies of CDI treatment in terms of cost utility performances [6–14]. These studies include an expression of the benefit to patients based on utility and quality-adjusted life year (QALY) measurement as defined by international recommendations [15, 16]. Utilities for patients presenting with CDI are poorly documented and are mainly derived from other conditions especially non-infectious diarrhea such as adverse events from oncology treatments [17] and chronic inflammatory bowel disease [18]. This study aims to provide a direct measurement on the utility decrement associated with the CDI infection in French hospital facilities.

## Method

### Study population

This is an observational prospective study performed in 7 French acute-care facilities located in Paris, Lyon, Lille and Valenciennes, 4 of which were large University Hospitals. The recruitment period was over the calendar year of 2016. Patient selection criteria were the following: adult (age > 18 years) inpatients presenting with a bacteriologically confirmed diagnosis of CDI during a stay in one of the participating hospitals for any clinical causes, accepting to participate and having given an informed consent. Patients presenting with chronic inflammatory bowel disease, involved in an on-going clinical study or unable to fill out a questionnaire for any linguistic or cognitive reasons were excluded. All cases were identified by the bacteriologists that established the CDI diagnosis in each participating facility.

### Data collection

The bacteriologist informed the interviewer in charge of the study of each case. The interviewer then met with patients to explain the study and ask for their informed consent in case they met the selection criteria. Patients were asked to fill out the EQ-5D-3 L [19] to describe their present state of health at the moment of the CDI episode (7 ± 2 days after diagnosis). Due to difficulties in arranging a new appointment with the patient after resolution of the current CDI episode because of hospital discharge or patient’s death, a retrospective assessment of the patients’ overall perception of health before the episode was also performed. Patients were asked to use the same instrument to describe retrospectively their state of health immediately before the present CDI episode (baseline). In the EQ-5D-3 L, the health status is described by ticking off one of three levels of functioning (“no problems”, “some problems” and “extreme problems”) on five dimensions: mobility, self-care, usual activities, pain/discomfort and anxiety/depression. Using a set of weights (value set or so called tariff) for each level of functioning in each dimension, the descriptive information can be converted into a single utility score. Since this study was conducted in France, the French value set was used to calculate utility scores [20]. Patients were also asked to rate their overall perception of health on the EQ-5D-3 L Visual Analog Scale (EQ-VAS), which ranges from 0 (worst imaginable health state) to 100 (best imaginable health state). All relevant clinical data were collected from the patients’ records: primary and secondary diagnoses as coded in the Hospital Discharge Database, length of stay, stay in ICU, immunosuppression, presence of any CDI episode within the previous 2-year period and all clinical data needed to characterize the severity of CDI episode. A classical measure of CDI severity is the Zar score which ranges from 0 to 6 (6 indicating the most important severity) [21]. This score is calculated by attributing one point to each of the following items: age > 60 years, temperature > 38.3 °C, albumin level < 2.5 mg/dL, or peripheral White Blood Cell count > 15,000 cells/mm3 within 48 h of enrollment. Two points are given for endoscopic evidence of pseudomembranous colitis or treatment in the intensive care unit. Patients with 2 points or more are considered by clinicians to have severe CDI.

Regarding comorbidity, a revised version of the Charlson index [22, 23], based on the ICD-10 coding of primary and secondary diagnoses recorded in the hospital discharge database was used. This index is calculated on the basis of the presence or absence of 17 comorbidities weighted according to their potential influence on mortality. Immunosuppression was considered present for patients with a concomitant immunosuppressive treatment; chemotherapy or radiotherapy; corticosteroid therapy for 30 days or more or at high doses (> 5 mg/kg prednisolone > 5 days); and/or presence of blood disease, metastatic cancer, or HIV infection with CD4 < 500/mm.

### Statistical analysis

Mean and SD for utility scores and EQ-VAS were calculated at the time of the CDI episode and at baseline. The decrement of utility scores for each patient was calculated by subtracting the current utility score from the baseline value. No adjustment for missing data in the EQ-5D was performed. It was tested whether the decrements of score were respectively dependent on the following categorical factors (Student t-test): age (< 65 years versus ≥65 years), gender (M/F), severity of the episode (Zar score ≥ 3 versus < 3), presence of previous CDI episodes (yes/no), Charlson index (< 3 versus ≥3), EQ-5D at baseline (negative versus positive), presence of immunosuppression (yes/no). A multivariate linear regression analysis was performed using the SAS (SAS V9.3) procedure PROC GLM to analyze the overall effect of these categorical variables. All variables were included simultaneously in the model without any option of automatic selection. The number of Quality Adjusted Days (QALDs) lost due to the CDI episode was calculated by multiplying the number of days from baseline (first symptoms) to the date of the interview by the decrement of utility. Quality Adjusted Years (QALYs) were calculated by dividing QALDs by 365.

## Results

### Population and CDI episodes characteristics

A total of 80 patients were included. Three patients died during hospital stay (for causes other than CDI) after having completed the survey and their data were included in the analysis. The population had a mean age of 69.4 years (SD: 14.5) and 55% were women. The characteristics of their hospital stays are presented in Table 1.

**Table 1:** Characteristics of the CDI episodes of the sample of 80 patients

	n (%)
Patients with a first episode of CDI Patients with previous episode (s)	68 (85.0%) 12 (15.0%)
Number of previous CDI episodes during the last 2 years (*n* = 12)	
Mean (SD)	2.0 (1.7)
Median / Min / Max	1 / 1 / 5
At least 1 episode in the last 6 months	9 (75.0%)
Presence of a pseudo-membranous colitis	3 (3.8%)
Treated in ICU (n, %) Death during the hospital stay (n, %)	2 (2.5%) 3 (3.8%)
Zar score of CDI severity	
0	13 (16.3%)
1	41 (51.3%)
2	15 (18.8%)
3	9 (11.3%)
≥ 4	2 (2.5%)
Presence of immunosuppression	29 (36.3%)
Number of stools per day at CDI diagnosis	
Mean (SD)	6.3 (3.9)
Median / Min / Max	5 / 1 / 20
Treatments prescribed for CDI (multiple responses possible)	
Metronidazole (n (%))	55 (68.8%)
Vancomycin (n (%))	27 (33.8%)
Fidaxomicin (n (%))	10 (12.5%)
Length of stay (days)	
Mean (SD)	30.3 (29.4)
Median / Min / Max	19.0 / 2.0 / 149.0
Time elapsed between admission and CDI onset (days)	
Mean (SD)	13.2 (19.7)
Median / Min / Max	4.0 / 0 / 109

The CDI was coded as primary diagnosis in 22.8% of cases indicating that it was the reason for the patient’s admission to the hospital. Other primary diagnoses were very diverse, the three most frequent being congestive heart failure (19 patients, 23.8%), chronic pulmonary disease (16 patients, 20.0%) and diabetes mellitus (14 patients, 17.5%). The mean Charlson index was 5.1 (SD: 2.8) with a percentage of 60% of patients being ≥5 indicating a high risk of mortality associated with severe comorbidities. Three patients died after a mean of 34 days (SD: 22.1) spent in hospital and 2 of them were treated in the ICU for their CDI.

The characteristics of the CDI episodes are displayed in Table 1. The CDI episode was considered as clinically severe for 32.5% of patients according to the Zar score (score ≥ 2). The number of stools per day may be also used as a severity indicator. At diagnosis, patients declared a mean number of 6.3 stools/day (up to 20 for one patient) and 88.6% of patients had more than 3 stools/day. At the date of EQ-5D-3 L completion, these figures were reduced to 2.4 stools/day in average and 38.5% of patients had > 3 stools/day.

### Quality of life and utility

The EQ-5D-3 L instrument, including the associated EQ-VAS, was administered on average 6.6 days (SD: 1.6) after CDI bacteriological diagnosis but 19.1 days (SD: 21.3) after the onset of symptoms (according to patient declaration), with a median of 12.0 days (min 3 days / max 124 days), among the 74 patients with no missing data. Additional file 1: Table S1 presents the answers to the 5 sub-scales as completed by the participants with the left column corresponding to the retrospective assessment of their health state before the occurrence of the current CDI episode (baseline) and the right column corresponding to their assessment at the day of interview.

The answers to the EQ-5D-3 L at baseline suggest that patients were already presenting with a severe condition having a substantial impact on their quality of life. A proportion of 34.5% of them reported at least one important or extreme problem (level 3) among the 5 dimensions of the questionnaire, especially anxiety/depression (12.5%), inability to perform usual activities (11.4%) and pain/discomfort (11.3%). This proportion increased to 68.7% during the CDI episode, with 5 patients mentioning the most extreme impact on all 5 dimensions. Pain/discomfort was rated extreme in almost half of the population (48.8%), inability to perform usual activities and confinement to bed was also very frequently reported (43 and 40% respectively).

Table 2 shows the utility measures obtained from the EQ-5D-3 L at baseline and during the current episode. Utility scores dropped from 0.542 (SD: 0.391) at baseline to 0.050 (SD: 0.404) during the CDI episode. Utility scores at baseline were negative in 14 patients (18%) with a mean of − 0.086 (SD: 0.075) and the number of patients that considered themselves with a perfect state of health (utility 1) was only 11 (13.9% of all). During the episode, utility scores were negative in 43 patients (54.4%) with a mean of − 0.266 (SD: 0.162) and positive in the rest of the population with a mean utility score of 0.427 (SD: 0.249).

**Table 2:** Utility scores and utility decrement

	Before the episode	During the episode
EQ-5D score (utility)		
Number of patients with no missing data (+)	79	79
Mean (SD)	0.542 (0.391)	0.050 (0.404)
Median / Min / Max	0.632 / -0.263 / 1.000	−0.052 / -0.530 / 0.888
Quartile 25 / Quartile 75	0.270 / 0.888	−0.293 / 0.365
Utility decrement associated with CDI		
Number of patients with no missing data (+)	78 (97.5%)	
Mean (SD)	−0.492 (0.398)	
Median / Min / Max	−0.476 / -1.416 / 0.750	
Quartile 25 / Quartile 75	−0.743 / -0.212	

(+) missing data in the EQ-5D filled out at baseline in one patient and during the episode in another patient

The mean utility decrement associated with the CDI episode measured in the 78 patients with no missing data was 0.492 points (SD: 0.398) with a maximum value of 0.750 and a minimum of − 1.416 in one patient (utility gain). It may be worth noticing that such a gain was observed in 5 patients indicating an improvement of their score at the moment of completion of the self-questionnaire compared to baseline.

At baseline, patients rated their state of health on the EQ-VAS instrument at a mean score of 61.0 (SD: 23.0), that dropped to 35.2 (SD: 19.8) during the episode i.e. a decrement of 25.8 (SD: 23.0) points.

### Factors associated with utility decrement

The utility decrement from baseline showed statistically significant increases according to the following characteristics of patients: Zar score of severity ≥3 (*p* = 0.001), a positive utility score at baseline (*p* = 0.032), being a woman (*p* = 0.041) and having an age > 65 years (*p* = 0.041) (Table 3). Conversely, the Charlson index, the presence of immuno-suppression or existence of a recurrence did not significantly modify this decrement. The same analyses performed with the EQ-VAS score were only significant for the Zar score of severity (*p* = 0.0189). The SAS procedure PROC GLM was used with the same variables on the utility decrement with a global statistical significance (*p* = 0.0025) (Table 4). It confirmed the previously documented results with the exception of the effect of gender which was no longer significant.

**Table 3:** Univariate analysis of the EQ-5D decrement and categorical variables

	n	Mean	SD	*P*-value
Gender				0.041
Male	36	0.393	0.380	
Female	44	0.577	0.397	
Age				0.041
< 65 years	32	0.380	0.412	
≥65 years	48	0.567	0.374	
Recurrence				0.893
No	68	0.490	0.399	
Yes	12	0.507	0.407	
Immunosuppression				0.234
No	50	0.539	0.374	
Yes	29	0.428	0.431	
Charlson score				0.937
< 5	32	0.488	0421	
≥5	48	0.495	0.386	
Zar score				0.001
< 3	69	0.434	0.383	
≥3	11	0.849	0.290	
EQ-5D at baseline				0.032
< 0	15	0.296	0.181	
≥ 0	64	0.539	0.421

**Table 4:** Regression analysis of patient and CDI characteristics with utility decrement as the outcome variable

Variable (reference)	Estimate	95% LL	95% UL	*P*-value
Gender (male)	−0.100	−0.2668	0.0664	0.2347
Age (≥65 years)	0.240	0.0229	0.4579	0.0308
Recurrence (No)	−0.042	−0.279	0.194	0.7217
Immunosuppression (No)	0.0072	− 0183	0.198	0.9398
Charlson score (≥5)	−0.076	−0.288	0.135	0.4730
Zar score (< 3)	−0.374	−0.609	− 0.138	0.0023
EQ-5D at baseline (< 0)	−0.210	− 0.423	0.003	0.0535

*LL* lower limit, *UL* upper limit

### QALYs lost due to CDI

Assuming that the health-related quality of life as measured by the EQ-5D-3 L was stable during the CDI episode from symptoms onset until the end of treatment, it is possible to derive from these results an estimate of the utility loss associated with a CDI episode. The result obtained in the 74 patients with no missing data was 10.22 QALD (Quality-Adjusted Life Days) (SD: 19.44) or 0.028 QALY (SD: 0.053). By restricting the calculation to patients with less than 60 days of diarrhea (*n* = 72), the results are 7.2 QALD (SD: 7.41) or 0.020 QALY. These estimates did not include the QALY loss due to the excess mortality associated with CDI which should be estimated from external sources [5].

## Di**s**cussion

This study is among one of the first attempts to directly measure the utility decrement associated with CDI among a sample of patients presenting with this infection. Another study was conducted in a UK sample of 30 patients hospitalized with CDI [24]. A different approach was used as the EQ-5D scores in the CDI patients were compared to the reference value for patients of similar age in the UK general population. The mean score index during the CDI episode was much higher in this UK sample than in this study (0.42 versus 0.05) suggesting that our sample of patients suffered from much more severe infections and/or more severe comorbidities. As very limited data were presented about the clinical characteristics of the UK sample, it is not possible to elaborate further on this comparison. Another striking obstacle in the comparison concerns the fact that different value sets (UK versus France) were used in these two studies. From a general methodological standpoint, it seems however questionable to directly derive a utility decrement of CDI from a comparison to a general population sample as it is highly uncertain to know whether this difference is attributable to CDI itself or to the effects of hospitalization and associated co-morbidities. The clinical characteristics of our sample suggested the presence of severe comorbidities even in patients hospitalized for their CDI episode. It is anticipated that the memory bias associated with a retrospective assessment of the EQ-5D at baseline had fewer limitations in this estimation than using a general population comparison [25]. Other observational studies have explored the importance of memory bias associated with retrospective assessment of Health-Related Quality of Life as compared to general population reference especially in the case of acute-onset injury or illness [26]. Even by using assessment over a retrospective period of 1 year, the authors suggested that retrospective evaluation of health status was more appropriate than the application of population norms to estimate health status prior to acute-onset injury or illness, although there may be a small upward bias in such measurements. In this study, the baseline health state, defined as the period immediately before CDI symptoms onset, occurred only 19.1 days on average prior to the date of the interview and this duration was not correlated with the utility decrement.

A specific CDI health-related quality of life instrument (HRQoL), adapted from the SF-36 instrument, has been recently developed but without results that could be translated in terms of utility decrement [27]. Such disease-specific instruments are designed to detect even small changes in the patient’s HRQoL. However, health economists prefer to use generic instruments because they enable the comparison of health states as well as the benefits of medical interventions across diseases. Most published cost-effectiveness models about alternative treatments of CDI have used proxies based on analog situations, especially GI tract diseases. A few studies have actually analyzed the HRQoL impact of GI tract disease with associated diarrhea. In Sweden, a study was performed in 116 patients presenting with collagenous colitis with the SF-36 questionnaire. Patients with active disease (≥ 3 stools/day or > 1 liquid stool/day) scored worse HRQoL than patients in remission [28]. Another study [29] was performed in 592 Inflammatory Bowel Disease (IBD) patients treated in six Hungarian tertiary centers using the SF-36 questionnaire and observed similar results regarding a significant association between the SF-36 score and disease activity (*p* < 0.001). Diarrhea was perceived as the most severe symptom during the course of the disease. Similarly, Stark et al. [18], in a German study using the EQ-5D on 447 patients presenting with IBD, showed that patients in remission had a significantly higher utility score than with active disease: 0.95 (SD: 0.08) versus 0.75 (SD: 0.25) in Crohn’s disease (*p* < 0.0001) and 0.96 (SD: 0.08) versus 0.84 (SD: 0.15) (*p* < 0.0001) in ulcerative colitis. Similar results have been published in patients presenting with Irritable Bowel Syndrome (IBS). In the study by Wang et al. [30], the mean EQ-5D index score was 0.739 for IBS and 0.854 for non-IBS. After adjustment for age, gender and presence of significant illnesses in another family member of the participant, the effect of IBS on the EQ-5D still showed a significant difference [− 0.11 (95% CI: -0.15 to − 0.07), *p* < 0.001]. In the Canavan study [31], the median overall utility score for the cohort before seeing a gastroenterologist was 0.76 (interquartile range: 0.69 to 0.80). These results are however not fully comparable to those obtained in hospitalized patients presenting with CDI occurring on top of severe comorbidities.

Another therapeutic domain of reference for this external comparison concerns the HRQoL impact of diarrhea as an adverse event (AE) of oncology treatments, a condition well documented in clinical trials with a standardized grading of severity [32]. A recent review was published [15] identifying two studies performed in the UK [33] and Japan [34] which included diarrhea in their scope. Both studies used short descriptions (vignettes) of the AEs of interest to obtain utility values for chemotherapy-related AEs by surveying small samples of the general public about their preferences (using a Time-Trade-Off approach) among the different health states described in the vignettes including the presence/absence of diarrhea of various severities. The results obtained in terms of utility decrement were in the range of 0.07–0.08. A similar study was performed in the UK among 100 members of the general public to elicit utilities for radioactive iodine-refractory differentiated thyroid cancer and evaluate the impact of treatment response and toxicities on HRQoL [35]. The authors found a mean utility for diarrhea of 0.42 (0.29) with an adjusted utility decrement of 0.47 (95% CI: 0.52–0.41). They noticed that it was perceived as the most burdensome health state among all other AEs presented. These values were however lower than previous estimates from vignette studies conducted in other malignancies. In metastatic renal cell carcinoma, a study reported a mean utility of 0.53 for grade III diarrhea [36]. Other studies in metastatic breast cancer and non-small-cell lung cancer reported values of 0.61 and 0.6 respectively [37, 38]. These differences may be the consequences of variation in the health state descriptions used between studies or differences in the population samples resulting in difficulties in comparing the values directly.

Another aspect that makes these evaluations difficult is that CDIs analogous to other clinical states with diarrhea are transient. This feature is important to consider in all studies performed by interviewing a sample of the general public and may generate a bias because methods designed to capture the preferences for transient health states may not conform to the axiom of constant proportional trade-off as the duration of the health state may affect valuation [39]. This limitation applies also in part in our study. This possible bias does not concern the patients with CDI who are fully aware of the fact that their infection is a transient state. It concerns the utilization of the tariff derived from a general population survey where the vignettes submitted did not include the dimension of the likely time spent in the health states. The negative utility decrement (utility gain) observed among 5 patients may be explained either by the improvement of their CDI symptoms, or by the resolution of their comorbidity or both.

The sample size of our study was limited due to the difficulty of implementing the protocol. Some of our results should therefore be interpreted with caution. This applies especially to our analysis about the factors affecting utility decrement. The absence of a statistically significant difference between patients presenting with recurrence or first episode could for example be explained by a lack of statistical power.

## Conclusion

The impact on quality of life of CDI episodes aside from the consequence of excess mortality is major and translates in a substantial QALY loss despite their short duration.

## Additional file

Additional file 1: **Table S1.** EQ-5D-3 L results (*n* = 80 patients) (DOCX 34 kb)
