# Supplementary material for: Quality of life and utility decrement associated with Clostridium difficile infection in a French hospital setting
Source: Health Qual Life Outcomes. 2019 Jan 11;17:6. doi: 10.1186/s12955-019-1081-5 (PMC6329091; doi:10.1186/s12955-019-1081-5)
Supplement: Supplementary file 1 — Table S1. EQ-5D-3 L results (n = 80 patients) (DOCX 34 kb) [file 12955_2019_1081_MOESM1_ESM.docx]

**Table S1. EQ-5D-3L results (n=80 patients) (supplementary data)**

|  | Before the episode | During the episode |
| --- | --- | --- |
| Mobility |  |  |
| 1 I have no problems in walking about | 39 (48.8%) | 11 (13.8%) |
| 2 I have some problems in walking about | 36 (45.0%) | 37 (46.3%) |
| 3 I am confined to bed | 5 (6.3%) | 32 (40.0%) |
| Self-care |  |  |
| 1 I have no problems with self-care | 53 (66.3%) | 17 (21.3%) |
| 2 I have some problems washing or dressing myself | 25 (31.3%) | 42 (52.5%) |
| 3 I am unable to wash or dress myself | 2 (2.5%) | 21 (26.3%) |
| Usual activities (e.g. work, study, housework, family or leisure activities) |  |  |
| Missing data | 1 | 1 |
| 1 I have no problems with performing my usual activities | 42 (53.2%) | 12 (15.2%) |
| 2 I have some problems with performing my usual activities | 28 (35.4%) | 33 (41.8%) |
| 3 I am unable to perform my usual activities | 9 (11.4%) | 34 (43.0%) |
| Pain / Discomfort |  |  |
| 1 I have no pain or discomfort | 30 (37.5%) | 10 (12.5%) |
| 2 I have moderate pain or discomfort | 41 (51.3%) | 31 (38.8%) |
| 3 I have extreme pain or discomfort | 9 (11.3%) | 39 (48.8%) |
| Anxiety / Depression |  |  |
| 1 I am not anxious or depressed | 33 (41.3%) | 23 (28.8%) |
| 2 I am moderately anxious or depressed | 37 (46.3%) | 38 (47.5%) |
| 3 I am extremely anxious or depressed | 10 (12.5%) | 19 (23.8%) |
| Number of patients with answers at level 3 (most severe) |  |  |
| 1 answers | 19 (23.8%) | 18 (22.5%) |
| 2 answers | 5 (6.3%) | 7 (8.8%) |
| 3 answers | 2 (2.5%) | 12 (15.0%) |
| 4 answers |  | 13 (16.3%) |
| 5 answers |  | 5 (6.3%) |
| Visual analog scale score of perceived state of health (0-100) |  |  |
| Mean (SD) | 61.0 (23.0) | 35.2 (19.8) |
| Median / Min / Max | 60.0 /0/100 | 30.0 /0/90.0 |
